# Option B+ Program for the Prevention of Vertical Transmission of HIV: A Case Study in Johannesburg, South Africa

**DOI:** 10.3389/fpubh.2020.533534

**Published:** 2020-10-28

**Authors:** Melanie A. Bisnauth, Ashraf Coovadia, Lawrence Mbuagbaw, Michael G. Wilson, Stephen Birch

**Affiliations:** ^1^Centre for Health Economics and Policy Analysis, McMaster University, Hamilton, ON, Canada; ^2^Empilweni Services and Research Unit, Department of Pediatrics and Child Health, Rahima Moosa Mother and Child Hospital, Johannesburg, South Africa; ^3^School of Public Health, University of Witwatersrand, Johannesburg, South Africa; ^4^Biostatistics Unit, St. Joseph's Healthcare Hamilton, Hamilton, ON, Canada; ^5^Department of Clinical Epidemiology and Biostatistics, University of Toronto, Toronto, ON, Canada; ^6^Taylor Chair and Centre Director, Centre for the Business and Economics of Health, University of Queensland, Brisbane, QLD, Australia

**Keywords:** HIV/AIDS, maternal health, PMTCT program, qualitative, accessibility, South Africa, patient-centered care

## Abstract

**Background:** South Africa's National Department of Health adopted WHO's 2013 consolidated guidelines on ARVs for HIV treatment and prevention in 2015, including changes for Prevention from Mother-to-Child Transmission (PMTCT) through *Option B*+, aimed to reduce the HIV prevalence rate amongst women by placing them on lifelong treatment, irrespective of their CD4 count. As a result, these guidelines were implemented for the PMTCT program at Rahima Moosa Hospital. Little is known about the impact of these guidelines on the work of healthcare workers (HCWs) and no research had focused on how these changes have affected adherence for the patients.

**Objectives:** The purpose of this research project was (1) to explore the impact of the Option B+ PMTCT program on the work of healthcare professionals, and (2) to understand pregnant HIV-positive women views and experiences with ART for life, as a way to better manage the Option B+ PMTCT program.

**Design:** Qualitative semi-structured interviews with a phenomenological approach was used.

**Setting:** Data collection at the antenatal/postnatal clinics/wards, OBGYN and Department of Pediatrics at RMMCH in Johannesburg.

**Method:** A qualitative study design is used with a phenomenological approach. The methodology used semi-structured interviews with healthcare professionals and patients. The thematic analysis was used within an Accessibility Framework to guide the identification of domains that emerged from all transcribed data. A convenience sample in the antenatal clinic, postnatal clinic, antenatal ward, OBGYN, and Department of Pediatrics and Child Health at RMMCH. The study is situated in Johannesburg, South Africa.

**Results:** The findings demonstrated that work has become difficult to manage for all healthcare professionals because of (1) the need for strengthening indicators for tracking to decrease loss to follow-up (LTFU); (2) inconsistency in delivery of counseling and support services and the need for communication across clinical departments; and (3) the lack of compassion and understanding by service providers. The difficult healthcare environment has affected overall views and experiences of pregnant HIV-positive women going on ART for life. All patient participants (*n* = 55) responded that they chose to take the fixed-dose combination (FDC) for life to protect the health of the baby and felt ART for life can be stopped after giving birth, unaware of the long-term benefits to the mother.

**Conclusion:** The Option B+ program emphasized a need for the provision of continuous counseling and support services for women with same day initiation of ART. There is a need for better internal communication and collaboration amongst HCWs across all units of RMMCH for attainment in treatment outcomes. HCWs communication to patients is essential in helping patients build trust in service delivery, decreasing the LTFU and promoting adherence. The ability to understand functions of the work environment in which a PMTCT program operates in is essential in addressing policy implementation and program issues for ease of adaptability of Option B+ programming on a larger scale across all units of RMMCH. Implications for future research include the need to address changes within the healthcare system at both clinical and management levels. It is crucial to incorporate the perspective of patients in policy implementation; uptake and adherence are key indicators in informing whether the Option B+ PMTCT program is being adapted into state hospitals effectively. There needs to be extensive research on how to strengthen indicators for long term scalability and sustainability of the program. Future evaluations need to address how interdisciplinary collaboration within healthcare facilities improves the management and understanding of Option B+ program.

## Introduction

South Africa (SA) had a significant number of 88,000 new infections amongst females between the ages of 15–24 years old in 2018 (1). People living with HIV (PLHIV) in SA accounted for 7,195,500 with a HIV prevalence for 15–49 year olds of 18.79% (2). Furthermore, the reproductive ages of 15–49 year old females account for an alarming 42% HIV prevalence in the country indicating that there is still major work to be done to help reduce the risk of transmission, especially amongst women and mothers (1). Furthermore, an estimated 70.4% of maternal deaths in South Africa were associated with vertical transmission of HIV infection (3). HIV prevalence in pregnant women attending antenatal care (ANC) remains extremely high and ~300,000 children are born to HIV-infected mothers each year across the country (4, 5).

The World Health Organization (WHO) (3) stated that prevention of mother to child transmission (PMTCT) of human immunodeficiency virus (HIV) is particularly a major issue. PMTCT is a clear example of a maternal and child health program that was offered across SA since 2001 (6, p. 118–120). The PMTCT cascade represents a complex system of sequential, interdependent steps that women living with HIV (WLWH) navigate as healthcare users to receive appropriate care and treatment for themselves and their newborns (7, p. 201–390). PMTCT typically entails women as healthcare users having access to services such as ANC, HIV testing, and counseling (HCT); prophylactic antiretroviral treatment (ART); education on safe delivery and infant feeding; follow-up HIV testing at a 7-day postnatal visit, and family planning (8, 9, p. 120–121). A vital component that was not traditionally part of the PMTCT program is the provision of life long antiretroviral treatment (previously known as Option B+) services for women as an addition aligned to the WHO consolidated guidelines (6, p. 121). Option B+ aimed to reduce the HIV prevalence rate amongst pregnant women by placing all pregnant women on ART for the rest of their lives, irrespective of their CD4 count, on triple ARV drugs in the form of a fixed dose combination (FDC) pill start the same day the patient is diagnosed as being HIV-positive (7).The FDC was introduced, made up of three drugs; tenofovir (TDF), lamivudine (FTC/3TC), and efavirenz (EFV) used in the first-line regimen which National Department of Health in SA adopted because it was simpler to manage, more effective, and cheaper to have women take only one pill once a day instead of three or more ARVs multiple times a day (9, p. 27–50).

Thus, the PMTCT cascade of care does not end at delivery of the baby as there is a need for long term follow-up. PMTCT using lifelong treatment is a proven, efficacious intervention, and can reduce vertical HIV transmission to <2% (7, p. 390).

In 2015, South Africa's National Department of Health (DoH) adopted WHO's 2013 “*National consolidated guidelines for the prevention of mother-to-child transmission of HIV (PMTCT) and the management of HIV in children, adolescents and adults”* on the use of ARVs for treatment and prevention of HIV infection across all age groups and populations, based on providing a broad continuum of HIV care across the country (3). The Government of South Africa had adopted these guidelines as a rapid approach to reduce new HIV infections amongst children by 90% and reduce the number of HIV related maternal deaths by 50% to strengthen the existing national healthcare system of Africa and improve its effectiveness (10, 11, p. 8–10).

The adaptability of PMTCT HIV programming is critical for reducing maternal and child mortality and morbidity rates (3). Adaptability is a concept that should be explored when looking at provision of PMTCT services in a healthcare setting where many women access care. We needed to better understand what components of Option B+ were working for women accessing PMTCT services. It is important to explore the concept of adaptability because there is a literature gap that exists when determining whether Option B+ is successful in policy implementation in terms of scalability and sustainability. Adaptability determines whether the program can be transferred between different population settings. For example, some studies emphasize the importance of evaluating the Option B+ PMTCT program in South Africa since there is not enough research for the long-term outcomes of this program (12, p. 179–193). Many HCWS have highlighted in key informant interviews that they cannot say whether it is effective in the long term (12, p. 179–193).

Therefore, the aim of this research was to understand both the experiences of the pregnant HIV-positive women who were placed on ART for life and the effect on healthcare service providers in the demanding public healthcare setting of the hospital that was first to implement Option B+ PMTCT in Johannesburg. The objectives of this study was; (1) to explore the impact of the Option B+ PMTCT-program on work of HCWs, and (2) to understand these patient experiences with lifelong ART, to better manage the program. We will answer the following research questions; (1) *What are pregnant HIV-positive women's views and experiences about lifetime treatment with ARVs and; (2) How has Option B*+ *PMTCT affected the work of HCWs?*

This exploratory study of Option B+ implemented at RMMCH will add to existing literature by providing research on the experiences of the PMTCT lifelong program roll-out and insight on women and their adherence to PMTCT programming.

## Methods

### Study Site

One of the regional public sector hospitals that was first to implement Option B+ PMTCT was Rahima Moosa Mother and Child Hospital (RMMCH), the study site that will provide insight to explore perceptions of women first going onto the lifelong treatment program. RMMCH provides healthcare specialist services for women and children. The institution has 110 general pediatric beds, 30 neonatal beds and a six-bed intensive care unit. The healthcare workers (HCWs) see more than 36,000 outpatients and have more than 12,000 births annually. Approximately 25 clinics refer patients to the ANC service at RMMCH, creating a busy maternal healthcare environment (11, p. 8–10). RMMCH is located in the suburb of Coronationville comprised of 2,500 people living in informal settlements of which 60.8% are females, many women with poor health outcomes (11, p. 8–10).

### Ethical Approval

Ethics approval for the study was granted by The Hamilton Integrated Research Ethics Board (HiREB) at McMaster University located in Hamilton, Canada. Authorization was obtained from the Human Research Ethics Committee (HREC) at the University of Witwatersrand, South Africa in 2015 and from RMMCH, Johannesburg to conduct the study in Coronationville (approval HiREB Number: 15-264-S/HREC Number: M150495).

### Design

This qualitative study conducted 67 semi-structured, audio-recorded interviews with pregnant HIV-positive women (*n* = 55) and HCWs (*n* = 12).

A phenomenological approach was used to investigate lived experiences of HIV-positive pregnant women and HCWs under the Option B+ PMTCT program. Patients and HCWs perceptions of HIV were investigated to learn more about their perspectives and stories prior to the implementation of Option B+. For example, patients were asked “*How did you feel when you first found out you were HIV-positive?”* Whereas, HCWs were asked, “*What are some of the challenges with adherence to Option B*+ *with your patients?*” This study explored the specific phenomenon between HCWs (nurses, physicians and healthcare management) and patients, providing in depth understanding of how the Option B+ program is now impacting the work of HCWs and the adherence of patients (13).

### Theoretical Framework

**Figure 1:**
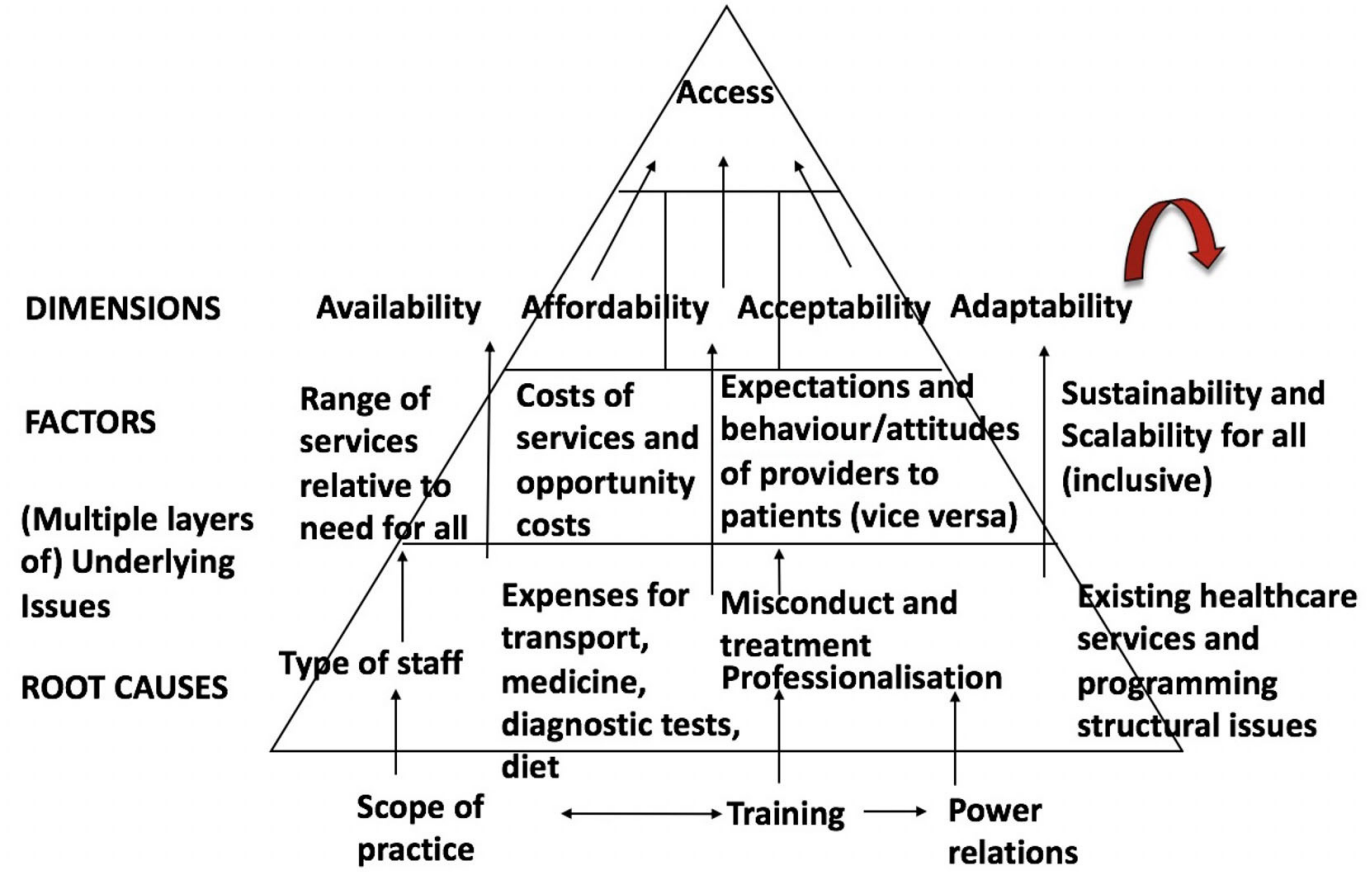
The enhanced accessibility framework [adapted from (12)]. The adaptability concept has been added to figure. Accessibility evaluation framework.

The Accessibility Framework (see Figure 1) has been adapted by McIntyre et al. (12, p. 179–193) for access to PMTCT lifelong treatment based on three dimensions of accessibility; affordability, availability, and acceptability to help guide the research. Each dimension represents a potential barrier to PMTCT care. However, the framework has an additional concept of “adaptability” of PMTCT services in healthcare settings. It is important to explore the concept of adaptability because there is a literature gap that exists when determining whether Option B+ is successful in policy implementation in terms of scalability and sustainability.

### Recruitment of Participants

Study participants were selected to gain a selected representation of views from the identified groups of; pregnant HIV-positive women and HCWs at RMMCH. Participants were not excluded based on race or language. The inclusion criteria included pregnant women between 15 and 49 years old, be HIV-positive and either newly enrolled or on Option B+. Newly enrolled HIV positive women on the program had to have at least their first dose of the FDC pill or been on treatment for at least one month and were included to provide insight on their experience with switching or starting on a new treatment regimen. HCWs had to have involvement with patients on Option B+ or/and the initiation of the program at RMMCH for at least the past six months since its inception (January 2015).

HIV-positive patients were recruited through convenience sampling, a non-probability technique by the principal investigator (PI) to select subjects because of their accessibility and proximity in the antenatal (ANC) and postnatal clinics (PNC) and wards, Obstetrics and Gynecology and, Department of Pediatrics and Child Health at RMMCH (14, p. 42–45). To control for differences in age and levels of education, scoping was completed within the ANC and PNC clinics and wards to obtain a sampling weight to account for the demographics of women at the hospital. Assistance from the nurses was provided, who would first attend to the patients, those who were on Option B+ were asked if they'd like to meet with the PI and informed that their participation was completely voluntary in the study.

Staff from selected units and departments, which included HCWs and executive management at RMMCH, were recommended by the Director of the Pediatric unit and contacted via email by the PI.

HCWs and patients were provided with information letters and consent forms for recruitment into the study. Individuals that provided informed consent were then asked to participate in an interview with the PI. Potential participants that were illiterate were provided an oral consent form and a witness to sign that the PI has read and clarified any questions.

### Sample Size

Participants were recruited consecutively until data saturation occurred where the PI is no longer hearing or seeing new information emerging from the data (14, p. 42–45). Thus, a total number of 67 interviews were conducted with 55 patients and 12 HCWs, which consisted of nine females (5 nurses, 1 pediatrician, 1 OBGYN, 1 neonatologist, 1 executive manager) and three males (2 pediatricians and 1 OBGYN). The reported mean for the experience in profession (number of years) for nurses is 21 years, pediatrician is 10 years, OBGYN is 6 years, neonatologist is 7 years, and executive manager is 8 years.

## Data collection and analysis

### Qualitative Semi-Structured Interviews

The interviews were scheduled during clinic hours for patients and working hours for HCWs. A small meeting room in the ANC was used for the patient interviews. HCW interviews were conducted in the privacy of their own offices. With permission, the interview was audio recorded and transcribed. Interviews were based on a discussion guide informed by the Accessibility conceptual framework. All participants' information including demographics, journal memos including observations of participant behavior during the interview was used with triangulation method in addition to the qualitative data entered using software QSR NVivo 10.0. Triangulation was used to enhance authenticity of the research process and findings, to ensure that different phenomenon of study participants were presented.

A record of the study was maintained through memos and journaling. The purpose of the memos was to maintain a process log and capture any observations at specific points of time from the PI, especially when conducting the interviews (i.e., body language of participants when answering questions). Furthermore, memos allowed the PI to maintain a record of analytic decisions and any alterations made throughout data collection and analysis. For example, behavior from participants was recorded to help open discussion and analyze certain themes that emerged and explore them further in depth. The written and electronic record from QSR NVivo 10.0 of the coding scheme and interpretative processes used in the data analysis included all files and any changes made to the database. Furthermore, the timelines for the study allowed the PI to complete tasks in a timely manner in order to allow time to cross check and ensure validity of the findings. A member check was completed with HCWs to improve validity of the transcribed interviews. In order to ensure accuracy and understanding of the patient participant responses, the PI would ask patients to reiterate for further clarification.

Patients were asked about their experience, knowledge and education of Option B+, trust of HCWs and, obstacles that had any impact with medication adherence (i.e., family obligations, number of dependents, difficult working hours, lack of understanding HCWs, lack of accessibility to the local healthcare facility, and lack of income). HCWs were asked whether they understood obstacles that impact adherence for their patients. Interviews allowed for both HCWs and patients to discuss certain aspects of their relationship such as trust and understanding of each other. The HCWs were asked information on their experience in relation to the Accessibility Framework to better understand their provision of Option B+, trust of their patients' medication adherence and, challenges they themselves encounter with their patients including awareness of opportunity costs their patients may undergo.

Data analysis consisted of comparing the transcribed interviews, literature, reflection, and continuous refinement to hone on most prominent themes and relationships. The research conducted inductive and deductive analysis simultaneously. Inductive reasoning allows for the PI to avoid separating stages of design, data collection and analysis but work forwards and backwards between data, conceptualization and period of collection (15, p. 341–360).

All electronic transcripts were transcribed verbatim by the PI. There was no translation needed, all participants spoke English. The process of data reduction began with open coding; all interviews were first transcribed during the interview and audio recording, then re-listened to and re-read to identify possible meanings and concepts throughout the data collected. In addition, a journal with notes was maintained to track the progress of the study and included memos related to the analysis of the data, including concepts, categories, or possible corroboration that was occurring at specific points in time throughout the data collection. The transcribed data were analyzed using a two-step method, first the audio recording and note taking was done simultaneously throughout the interview process and, secondly the audio recording was replayed and transcribed.

A thematic analysis was used with the Accessibility Framework to guide the identification of domains that emerged from all transcribed data including the audio recorded interviews and transcripts with the help of QSR NVivo 10, software, a qualitative data program that helps organize the volume of data was used to help draw relations and common themes throughout the data that was entered and analyzed using the Accessibility Framework.

The domains of interest that were explored included the following; stage of pregnancy; initial interaction with Option B+; adherence history; challenges with FDC such as stigma; acceptance and management of regimen; availability of PMTCT services; affordability including opportunity costs of missed time from work to access care; social and cultural barriers; relationship with HCW; and knowledge of Option B+.

The PI and research supervisor informally compared perceptions of the data and contributed to the coding scheme. Transcriptions were coded separately and a triangulation technique was utilized to cross verify data sources. To ensure the credibility of the findings, a secondary analyst reviewed the findings and coding from transcribed interviews.

The second phase of analysis used axial coding. The observations and any questions that were recorded in memos assisted in developing of an initial coding scheme. Consensus was then achieved on the third version of the coding scheme. These major categories were aggregated from the raw data collection and then QSR NVivo 10.0 was used to reduce and identify themes across all sources of data. Themes that were identified were compared to determine relevance and value in describing the Option B+ PMTCT implementation at RMMCH.

## Results

### Demographics of Patients and HCWs

Self-reported demographic data was collected from 67 participants which included 55 HIV-positive pregnant women from the ANC maternity and PN wards and 12 HCWs including nurses, Pediatricians, Obstetricians and Gynecologists, Neonatologists, and managers. The average age of the HCW was 45.6 years old. In addition, the average years of work experience reported by HCWs in their current occupation was 13.5 years and consisted of 75% female and 25% males.

The average age of a patient participants was 33 years old; most women had a total of three dependents and had undergone at least one still birth. For the total of 55 patient participants, 36 were not married. 34 of the 55 patient participants were on Option B+ for six months and only 2 patients on Option B+ for less than a week. The average duration of interviews was one hour with participants.

The findings revealed that the implementation of the Option B+ PMTCT program according to the national consolidated guidelines has been challenging for both patients and HCWs. The following four sections discuss the findings of how the program impacted the healthcare environment for (a) patients; (b) frontline nurses; (c) physicians; and (d) managers for each of the four dimensions of access, *availability, affordability, acceptability*, and *adaptability*.

### Availability (Physical Access): Is the Quality of Care Diminishing?

Availability can create differences in access to services such as, the provision of counseling and support services for the Option B+ program may be sacrificed as a result of resource constraints (12, p. 179–193). Availability is concerned with whether appropriate healthcare providers or services are supplied in the right place and time to meet the needs of these HIV-positive pregnant women including; hours of operation, drug supply of ARVs, and willingness of service providers (12, p. 179–193).

#### Patients: Education—Is It Beneficial or Harmful?

Pregnant women feel the need to adhere only until giving birth, not understanding that ART is beneficial in the long term. Many of these women feel the need for more education to understand why they are taking this drug beyond childbirth. A 38 year old patient, married and had undergone three miscarriages stated,

*I don't feel like there is enough education here at RMMCH. I have gone to my local health clinic and they provided a three day workshop. I have done adherence events which were three hours each, teaching me about how to use ARVs*.

The lack of PMTCT knowledge posed a barrier to retention for the patient in understanding benefits of medication adherence. Knowledge translation should occur from the healthcare provider to the patient, but often it is impeded by unequal power dynamics causing the patient to feel intimidated or nervous to be proactive and ask questions. Patients during counseling sessions may view HCWs as authority figures. Many women reported it was difficult to understand what the nurses told them at times because it was unclear. Some women expressed that it was hard to ask questions in front of their peers and preferred to wait until they could do so in private. Whereas, a 35 year old patient and 8 months pregnant states,

*I have participated in education workshops here at RMMCH and find it useful. I learned about using condoms when having sex, eating healthy food and to not just sleep around without protection. My local health clinic does not give me as much information. Though I feel comfortable asking questions at the clinic and it is not as far, I come here to receive more education*.

Education and information is not consistent across the local health clinics for Option B+ PMTCT which can be harmful when only parts of the educational messages get across to patients. Patients have become overwhelmed with information they receive from various sources which included local health clinics, healthcare workers, media sources, and family/friends.

#### Frontline Nurses: Issues Arising With the Available Information of Option B+

There is inconsistency in delivery of counseling and support services for patients at RMMCH. There is a lack of; communication across clinical departments because guidelines are taking time to be translated across the healthcare system in a manner that allows for consistency in messaging; compassion and understanding by service providers due to the emotional risks that HCWs are undergoing associated with changes to their work. The implementation of the new guidelines for the Option B+ PMTCT program had created frustration for both clinical and management levels at RMMCH. One nurse stated issues faced with communicating updated information because of the constant changes to the new consolidated guidelines,

*Women are being educated. I think the problem is with all the changes we have to update our patients. There needs to be more education with the healthcare workers, then that gets transferred to the patient effectively. This way the patient is updated, and education can be emphasized. The woman cannot say they do not know. All sources they access need to align with the information they provide*.

#### Physicians: Support Is Needed

The RMMCH environment is very busy and there is a high demand for physicians to deliver good quality services. HCWs often transfer patients to different departments and units in order for the patient to receive better care and expertise for specific services they may need. Often, due to the busy environment, good quality services become sacrificed at times.

Physicians expressed how by the time the patient sees them, they understand that the patient had undergone testing by nurses and have had to go through the shock of findings out their diagnosis all in one day. By the time the physician speaks with the patient, they found them to be passive because they are complete strangers. Some physicians take it personally while others understand it is a process. One physician states, “*Healthcare workers don't understand at times; the patient has to be counseled repeatedly and in detail. It happens three times before the patient understands.”*

Physicians express that there needs to be support groups for women to be able to open up and communicate what they are feeling, “*We use to run an important support group in the clinic on Fridays. Women liked being together, now they just sit in the general public.”*

Findings suggested there was insufficient time and counseling services provided for pregnant women to absorb the shock of finding out her HIV diagnosis and starting lifelong treatment immediately. Option B+ had reduced the number of missed dosages for women through a one pill a day regimen. However, there was a lack of knowledge translation between HCWs and patients in understanding the benefits of the FDC. This posed a barrier to effective uptake in treatment and increased loss to follow-up.

#### Managers: Knowledge Is Power

The executive management level expressed that knowledge is power, women need to understand the benefits of lifelong ART in order to continue treatment. It is difficult to know whether patients are actually taking the medication because they are being told to do so or they actually understand the benefits clearly. This relied on knowledge translation from the HCWs consisting of both frontline nurses and counselors and healthcare physicians. An executive manager stated,

*The system is designed for doctors to be paternalistic to their patients when it comes to involving the patient in the treatment decision making process. This means no patient involvement, choice or patient authority- to enforce one direction. Partially because the pregnant HIV-positive women we see at the ANC, at no fault of their own, do not have education and little knowledge about what they are on. It is unfortunately the system that perpetuates the lack of patient involvement while they sit there waiting*.

RMMCH PMTCT committee had initiated administrative practices to help manage Option B+, which had challenges in education and awareness of ART and its integration into the already existent stigma and cultural beliefs in the surrounding South African community.

#### Monitoring and Evaluation of the PMTCT National Guideline Indicators

Management had utilized strategies that include education and training across departments that need to assist HCWs in better understanding the implementation of the program at RMMCH. There was still a need to strengthen indicators of the program to increase numbers of women on same-day initiation (placed on ART the same day as being diagnosed HIV-positive) in order to evaluate its success.

The development of the new national consolidated guidelines for specifically PMTCT promoted the proliferation of numerous policies and protocols. This led to developing indicators within RMMCH to help monitor and evaluate the Option B+ PMTCT program. The revision for strengthening these indicators is ongoing. Indicators were recorded monthly at the ANC and daily registers are used to record patients. Reports will be done monthly and annually for total births in the facility, maternal health, immunization, and HIV counseling and testing (HCT). The ongoing changes to indicators and recognizing gaps in monitoring and evaluating outcomes of the Option B+ PMTCT program had become challenging for RMMCH. A manager stated,

*I am finding a big gap and it is quite scary what education is being told to our patients at times, they are falling off the grid and incorrect information is an example of the issues with the education component of PMTCT program. The first person that speaks to a patient or the first interaction that patient has with information is what they will believe. It is hard to come in and now say something different to them. Incorrect information can act as a barrier*.

There are no established indicators, causing inconsistency in measuring the effectiveness of Option B+. This in turn, impacted the clinical training of HCWs with their delivery of the PMTCT program to patients at RMMCH. HCWs and management found it challenging to monitor the ongoing progress of Option B+ PMTCT for reporting at RMMCH. One administrator in the Empilweni HIV clinic emphasized issues with indicators that were arising,

*The WHO created these guidelines with unrealistic indicators that do not address certain gaps. For instance, say you have one hundred pregnant women being tested for HIV, out of those hundred women, say there are 10 positive babies delivered. There isn't any indicator that has been developed to tell us that those 10 babies belonged to 10 positive women from those 100 that were tested. There is a huge loss to follow-up with these women. Therefore the outcome of what is measured is not telling us the entire truth*.

#### Time Is Money

Often HCWs were under pressure due to the high demand of patients at RMMCH. Managing time is a challenge for HCWs and it had unfortunately caused them to deal with patients inappropriately impacting the quality of care provided. A manager stated,

*We are not aware much of the complaints from the community we serve here at time. Once patients get here, the treatment is not exactly friendly from the nursing staff. It is a struggle to access our hospital sometimes for these women and I know clinicians are poor at identifying patients due to the high demand of patients that we see here. Our catchment area is not geographically large and I was not aware patients bought addresses. Pretty sure that someone who comes in may have a difficult time getting here but will keep silent about it*.

### Affordability (Financial Access): Multiple ANC Identification Cards

Affordability is concerned with the individual's ability and willingness to pay in order to access healthcare services which can be measured by opportunity costs where patients can or cannot afford to take time from work to get to the hospital (12, p. 179–193).

#### Patients: Opportunity Costs

Many patients expressed how balancing their daily duties of being a mother and wife was challenging with adhering to their ARVs and attending their local healthcare facilities or RMMCH. For instance, some patients were working but had to take time off work to make appointments at the hospital or return for follow-up. Women reported that sometimes they have a hard time with their managers, even though they provided a doctor's note to their employer. Patients expressed concern over their lack of income which makes it difficult for them to travel to RMMCH. One pregnant HIV-positive woman stated,

*It is hard to get the taxi to go get the medication at times, I have to miss work and tell my manager I have to get medication. I get paid sometimes if I miss work, depending on if I provide a letter from the doctor. The little bit of money I make is only for groceries…it is very hard because I have my childrens' school fees, transport and other commitments…their father is not active*.

When patients were interviewed about if there were any challenges in retrieving ARVs, many would respond that it was “easy” to retrieve medication but explained they wait an average of 3 h. They would wake up early (before work if they are employed), rush their children to school and then wait in line to get their treatment. A 35 year old woman and thirty one weeks pregnant responded,

*I get my medication at the hospital and clinic. It is easier to get meds from hospital. I make an appointment to get it. I have to wait long to get medication, maybe three hours or more. I have to be early to get through the line-up fast. I am working but it depends on the price,. I don't think I could afford the medication but if I had no choice then I would have to pay and be willing to do so…On your side here at RMMCH, it is not as long and I don't have to pay for medication*.

#### Frontline Nurses: Multiple Addresses

RMMCH had many patients that do not live in the catchment areas that the hospital serves. Often a patient would enter the interview room carrying her ANC card which provided the name of the local health clinic in which she may be referred from in order for the HCWs to understand if the patient is within catchment areas of RMMCH. Frequently, the address that she provided was different from the one written on the card, while in some cases the card was photocopied or was not authentic. Often, women were unable to afford the transport home from RMMCH. A nurse commented,

*We provide money out of our own pockets to patients, so they are not left stranded at the hospital…It is the governments' duty to supply patients with medication. Our patients do not have medical aid, and most are not working. They lie about their addresses and if they do not live in surrounding areas, they have to go to the closest hospital where they stay. These pregnant HIV-positive women end up buying addresses for proof of residence and will pay community members in the squatter camps behind the hospital*.

Unsuccessful tracing and LTFU were mostly due to incorrect addresses received, likely from women giving false addresses due to fear of stigma and discrimination associated with using their local facilities. This phenomenon exists as a way for patients to be referred to RMMCH as a specialized hospital. Patients may not attend their local health clinic and be staying with a partner in the squatter camps nearby the hospital. If RMMCH is their closest healthcare facility and the patient does not want to pay for private services then buying an address may appear attractive to patients.

#### Physicians: Understanding Opportunity Costs That Our Patients Face With Option B+ PMTCT

The work environment had become less effective with managing the large number of patients. The physician's role had changed from providing support for the patient to spending less time managing patient adherence and conveying the importance of benefits of lifelong ART. Option B+ had decreased the amount of time physicians spend with patients by diagnosing and initiating them on treatment the same day. Physicians were unaware of the opportunity costs pregnant HIV-positive women undergo to attend RMMCH. One physician states, “*None of the patients really complain if you don't specifically ask them. Once I know one is stable on the regimen, I don't function for after hours for their problems.”*

#### Managers: ART Is Affordable

Executives perceived there was a major issue with affordability for these HIV-positive pregnant women when it came to ART. Managers felt that women have no choice but to have to take time off work and wait in the ANC for long periods of time. One executive manager states, “*Getting to the facility is needed, a woman's employer is not very sympathetic and if it isn't a sick day then they cannot afford to pay them. It has to come from their annual leave. This is one of the financial barriers these women are facing.”*

### Acceptability (Cultural Access)

Acceptability is concerned with the fit between provider and patient perception toward and expectations of each other, including perceptions toward characteristics of age, gender, ‘race’ or ethnicity, and language which impacts the relationship of delivery and recipient of care (12, p. 179–193). Respect and the ability to listen to symptom descriptions, undertake a thorough examination, explain illness and discuss treatment alternatives conducted by the healthcare provider especially at the first point of contact is important (12, p. 179–193).

#### Patients: Do HIV-Positive Pregnant Women Think/Feel They Have a Choice?

All 55 patient participants responded that they chose to take the FDC to protect the health of the baby and felt that treatment could be stopped after giving birth. They were unaware of the benefits of continuing treatment which is the largest barrier for effective initiation of Option B+. At times, the use of the word “Option B+” would confuse pregnant HIV-positive women thinking it wasn't necessarily “a must” stick to regimen for life. A thirty-two year old woman, married with three dependents responds, “*I feel like I have no option. I do this for the sake of the baby. I don't want to lie but I am bad with tablets. I care about the baby and don't want to play with its life and mine. I am responsible for another life.”*

Patient acceptability of medication adherence and the changes in their ART regimen was influenced by the information they received about safe sex practices, serodiscordant relationships, breastfeeding, family planning, and child spacing that contributed to difficulty in adherence amongst pregnant HIV-positive women.

#### Unsafe Sex Practices

Forty three of the pregnant HIV-positive women interviewed thought that having unprotected sexual intercourse with a partner that was also HIV-positive was fine as long as they were both on ARVs. These women did not understand and were not aware that re-infection can occur. Patients thought that taking ART could mean engaging in unprotected sex and did not understand why their viral load was increasing whilst on treatment.

When these women were asked if they are sexually active, many would say they were not and then when proceeding with the interview, they would state that they engaged in sex last week. There was uncertainty in responses when asked if a method of prevention is used and how often. One 29 year old woman, not married and 36 weeks pregnant with her second child stated,

*I am sexually active; the last time I had sex was two months ago. I use a condom every time and I don't want to make any mistake. I am now pregnant with this child…I don't know what happened, maybe the condom broke… I was having problems with my boyfriend. We would always use a condom, but for this pregnancy during the intercourse it was taken off. I felt like he knew something and took it off, always coming up with excuses during sex…like how he has a rash*.

#### Serodiscordant Relationships

When conducting interviews with the women, there was tension at times in the room when the question of, “*Is your partner aware of your status?”* was posed. Many women would respond that they have multiple partners but are not exclusive. A 40 year old woman, not married separated from her partner and is 32 weeks pregnant stated,

*I am not sexually active, fortunately. The last time I was sexually active, I was still together with my partner, who knows I am pregnant. A condom was used regularly, except the first time when we met and then I fell pregnant. I cannot say I regret it; I am an adult and responsible for my actions… My partner knows I am taking the ARVs and he is angry at me actually. He says it is going to take time because he is HIV-negative and asked for some time to go and heal*.

Furthermore, women disclosed at times that their spouse was HIV-negative and did not know she had engaged in sexual intercourse many times without use of a condom or other types of protection.

#### Breastfeeding: Miscommunication of Messages

A serious concern with misunderstanding the program by patients was discovered when majority of pregnant women it was not a risk to stop their ART during breastfeeding after delivery. Women would sit in the waiting room of the ANC and hear education sessions, but most importantly did not actually understand the messages communicated to them entirely. This aligned to data collected that woman's body language, confused facial expressions, twisting of the body, and scratching of the head demonstrated uncertainty.

When participants at times were asked, “*Did you attend any of the education sessions in the waiting room ran by the nurses?”* Many participants would respond, “*Yes*.” It is one thing to attend the information sessions that are provided in a lecture style format from the nurses in the waiting room- but to actually listen, understand and actually apply what is being taught to the patient was a challenge. When patients were asked, “*What did you learn from the education given in ANC?”* Many participants state, “*Breastfeeding when the baby is born is good…Is it true I can do that? [Only on ARVs]…oh…” or “I learned that I can breastfeed regardless of my status.”*

HCWs communicated messages to the patients but sometimes the patient will only understand part of the message which can be more harmful in the end. If the patient decides to breastfeed, they need to be carefully pre-counseled about adherence to ART and exclusive breastfeeding. At the time, post breastfeeding and 18 month HIV tests should be done as well. If the patient has a viral load of <1,000 copies/ml then the baby should preferably not be breastfed and infant formula can be substituted.

#### Family Planning and Child Spacing

There is a large dependence on partner(s) for support from a large number of pregnant HIV-positive women interviewed. When questioned, “*What does your partner think about you taking ARVs?”* A 26 year old woman revealed,

*I had sex about three days ago. Sometimes I use a condom and now that I am drinking medication around my partner I am more careful and make sure to use a condom…My partner does not know what my treatment is for and he sees me drinking it. They say I must not tell the partner and they may not like that I have HIV. This baby was not planned and I do not want to lie to you. I will tell him after at the clinic and disclose the status after the birth of the baby*.

Many of these women were unable to disclose HIV-status with partner and feel the need to hide their ARV medication. This is largely due to the fear of losing their partner(s) and not having support to provide for their dependents and newborn child.

RMMCH focused largely on mother and child with its delivery of Option B+ PMTCT. It had caused an issue with excluding the important male figure in family planning methods. Therefore, if a woman received her HIV-status and information is provided to her about disclosing to her partner(s), there is a barrier for the family unit to progress further because psychosocial support and adherence counseling services along with family planning would not be provided to the male counter-part.

Patients on average had three dependents and one still birth. One woman stated,

*I am 37 years old and not married but I have a partner. My first child died in 2007 and it was a still birth. I did not get to keep him at birth. I have been pregnant a few times, all died and one even buried at the age of five. I am 5 months pregnant with my next baby; this will hopefully be my first living child*.

An issue that presented itself was that women are likely to stop treatment in between pregnancies. Child spacing needed to be addressed in educational sessions with one-on-one counseling to these pregnant HIV-positive women for their own health. It is crucial in order to reduce the risk of transmission between mother and child, especially if she is not practicing safe sex and not using protection in between births.

#### The Fixed Dose Combination (FDC): More Manageable?

Patients found swallowing the FDC pill difficult in the first two weeks of starting ART or switching to Option B+. Patients found it easier to manage one pill a day after two weeks. There was an average of 0 to 3 missed dosages for women that were on FDC over a period of 6 months.

Patients found that taking the FDC reduced stigma at home and in the community because it was easier to hide one pill bottle in their purse than have many multiple bottles of ARVs. Women found it was easier to manage one pill and take it at night before going to bed.

However, all patient experienced side effects such as dizziness, tiredness, nausea, and vomiting but on average felt better on the one pill a day after the first two weeks when starting the FDC. When patients were asked if anything changed in their ability to take the medication, many responded that the pill has become part of their daily routine. For example, one woman, 35 years old, not married and 39 weeks pregnant with her second child responded,

*The pill may be really big and hard to swallow at first. I never miss any dosages –maybe with time sometimes because I drink it at night and usually take it at 9 p.m. and sometimes I am a few minutes late. I think I would go crazy if I missed it because I have made it part of my life*.

Furthermore, many of these women expressed that they were shocked at first finding out their HIV status, but those who felt comfortable explained that they had a support system. A 35 year old woman stated,

Yoh! I felt I was about to die and the world was ending. I was angry and confused and even blaming myself. I wouldn't say I was depressed and I always find someone to talk to -like my mother. I trust her the most. I didn't go through denial, I accepted it and moved forward and why, is because I suspected my partner and it was that kind of situation. I wouldn't blame him and I didn't know if I had it. I just want to see my life go on and I don't know if I am angry still, I just want to move on. I panicked at the moment and felt like killing myself, and I want to beat this. I am not sure if I want a partner.”

Pregnant HIV-positive women were not given adequate time to absorb the shock of their HIV status which is challenging for uptake of the program. They underwent testing, disclosure of HIV status and were initiated onto treatment for Option B+ all in a day. These women were bombarded with a large amount of information and needed time and guidance to adjust for going onto lifelong treatment.

#### Frontline Nurses: First Point of Care

Frontline nurses were the first HCW that a pregnant HIV-positive woman comes into contact with at RMMCH. Nurses each attend to an average of 21 patients a day. Many of the pregnant HIV-positive women nurses see a day had been on Option B+ for a maximum of 6 months. These nurses usually work with patients for many reasons, some of which include; vital sign tests; blood tests; medication distribution; non-stress tests; monitoring heartbeat; patient comprehensive reporting; make beds; urine tests; temperatures, etc. Therefore, there has to be acceptance from the frontline HCWs in understanding the new guidelines in order to correctly provide information to their patients.

Nurses gave women the opportunity to ask questions and tell them that they should feel free to come and ask any questions they may have and can do so in private. One nurse stated what she does to provide the best possible care to her patients,

*I teach them and tell them the importance of why they are attending the ANC and education, making sure they know why they take the medication. If she wants to speak to you privately, you listen to her and give her the best advice you can. It's hard you aren't with her 24-7, once she goes home it depends on her*.

Many nurses think that Option B+ is effective because the FDC is easier to manage as one pill instead of taking three ARVs. Patients are doing better on one pill that have children dependents because it is one ARV dosage in the evening and able to send their children to school and go off to work. Nurses found more women were accepting the FDC, though some were reluctant because they were scared of the side effects and the stigma that still exists about HIV/AIDS. One nurse expressed, “*Most women stick to the regimen; some are in denial. Adherence is increasing, unless they are telling us lies. When I speak with a patient, what they tell us we have to believe what they say about their adherence.”*

#### Physicians: Inconsistency in Thorough Counseling and Follow-Up

Physicians recognized that one of the biggest challenges is the opportunity for proper counseling that could influence pregnant HIV-positive women to make informed choices about treatment for life. Pre-therapy counseling is missing by definition of Option B+ PMTCT. The difference between Option B and Option B+ is that under Option B, the woman had the opportunity to digest the diagnosis of HIV and to decide whether she wants to take the ARVs for life, until she gave birth, or until the time she stops breastfeeding but with Option B+ though that choice is eliminated. This was overwhelming for an increased proportion of women not knowing their HIV positive and on the same day prescribed antiretroviral therapy for life. There was clearly an opportunity for more counseling with the Option B+ PMTCT program.

HIV-positive pregnant women had difficulties in understanding immediate ART initiation. Similar views on same-day testing and initiation on ART and viewed same-day testing and initiation on ART as “too rapid” for clients to be able to process. One healthcare worker in the OBGYN department stated,

*A woman, a mother comes into the hospital and may enter our antenatal clinic thinking she is just fine and healthy. Then a nurse asks her, to run a test, after of course waiting approximately from 6 a.m. to mid-day. She is tired, exhausted and carries on to pre-test thoroughly. Her test results come out as HIV-positive after cross-checking, the counselor now sees her for post-test counseling…Keep in mind now that she has not even seen the doctor yet. She waits to hear that she has an HIV-positive status. Now she may be very upset, not know how to register what is going on, and barely has any time at this particular moment to process before she is given an FDC pill and told that this will help her…whether or not she fully understands the benefits, who knows*.

This had caused inadequate space for counseling, and long waiting periods to see a HCW. The patient is rushed for testing and treatment which deters the patient from returning. At RMMCH, women still had to visit multiple departments in a single day with long waiting times. This can overall impact the patients experience and acceptance of Option B+ PMTCT and her ability to return for future follow-up appointments.

#### Managers

Management had noticed a major issue with pregnant HIV-positive women with adherence declining after giving birth. An executive explained why this may be,

*Women are motivated to take these medicines because there is a purpose to it during pregnancy, to prevent transmission for their unborn baby. Once the baby is out, that purpose becomes lost and there may be no incentive to maintain adherence*.

### Adaptability (Contextual Access): Is the Option B+ PMTCT Program Scalable Outside RMMCH?

Women participating in the Option B+ program are sources of valuable information that can help inform the Option B+ policy. However, there is failure across the current literature specifically in South Africa to involve patient perspectives. Research has found that the aspirations of increasing adherence may be more difficult than expected by key stakeholders (4). This may be a sign that the adaptability of a policy is dependent on all actors involved in Option B+ including the perceptions of the healthcare workforce, and the HIV-positive pregnant women.

#### Frontline Nurses

Frontline nurses suggested that scalability of the program outside of RMMCH may require more research in adapting the program to other healthcare facilities. This would mean system planning and ensuring that the program is sustainable. A nurse commented, “*It is going to take time and way more research to understand whether the program can scale-up outside of RMMCH. All we know from research is that pregnant mothers are defaulting…”*

#### Physicians

When physicians were asked about whether the Option B+ PMTCT program would be effective, many could not comment because of the lack of long term data about sustainability and scalability of the program. One HCW in OBGYN unit stated,

“*It is very difficult to evaluate the long term sustainability of the Option B*+ *program. I cannot comment even on the effectiveness of Option B*+ *in the short term. I have to look at long term data that further reduces PMTCT and it is currently not available for Option B*+*. This program was only introduced in January this year at RMMCH and I don't think there is sufficient data yet for South Africa.”*

#### Managers

The perception of an executive that manages the Option B+ PMTCT program at RMMCH aligned with much of the HCW views, that there was insufficient data to analyze whether the program would be scalable outside of RMMCH. RMMCH is still undergoing program implementation issues and would need to strengthen its system planning model before rolling out of the program occurs. A manager stated,

*As a project manager for PMTCT, my main function is to make sure the program is running well and implemented properly at the facilities…We have only introduced Option B*+ *for five to six months now. To be honest, I can't really say if it is really working because there has not been any evidence*.

## Principal Findings

### Patients

The findings suggested that patient acceptability of medication adherence and the changes in their ART regimen is influenced by the information they received. The quality of care made available to patients can affect their overall experience which contributed to decision-making for a patient. In addition, patients' willingness to incur opportunity costs and return to RMMCH was largely dependent on their experience. Patients require education and continuous support to understand the benefits of Option B+ PMTCT and ART for life. All 55 patient participants responded that they chose to take the FDC to protect the health of the baby and felt that treatment could be stopped after giving birth, unaware of the benefits for the woman to continue treatment.

### Frontline Nurses

The findings nurses suggested for nurses that the Option B+ PMTCT program had created emotional risk factors for frontline staff. Until frontline nurses are accepting of how their work had changed then the patient experience may be undermined. The initiation and maintenance of the program had increased responsibilities for HCWs in patient care which include; good quality care, delivery of educational sessions, communication and consistent messaging for patients, monitoring ART adherence, and the provision of thorough counseling.

### Physicians

The findings suggested for physicians that the healthcare work environment had become more ineffective with managing the large demand of patients. The physicians role had changed from providing available support for the patient to spending less time managing patient adherence and conveying the importance of benefits of ART for life. Physicians are unaware of the opportunity costs pregnant HIV-positive women undergo to attend RMMCH. However, increased access to ART does not resolve internal issues with HCWs. There are issues with ART initiation, viral load monitoring, thorough counseling and support groups, communication between department transfers, privacy, and overall quality of care for patients.

### Managers

The findings suggest that the hospital organization had initiated positive administration practices to help manage transmission of HIV in PMTCT. Option B+ has challenges in education and awareness of ART and its integration into the already existent stigma and cultural beliefs in the South African community. The executive staff had utilized strategies that included PMTCT HIV strategies. Educational lectures and training across departments need to assist HCWs in better understanding the implementation of the program at RMMCH.

There was still a need to strengthen indicators in order to better manage the implementation of the program. RMMCH had an increasing number of foreign national mothers that use the facilities up until they give birth which had contributed to the inability to trace a patient. There is a heavy burden of maintaining up-to-date tracking information with paper-based systems and lack of telephone numbers in records and unclear addresses. Studies suggest that tracing should be done the first week once a patient misses an appointment because it is the most critical period, since after treatment is initiated it is crucial that the woman remain on ART (16).

## Discussion

The research demonstrated that work had become difficult to manage for all HCWs because of the; (1) need for strengthening indicators to decrease loss-to-follow-up; (2) inconsistency in delivery of counseling & support services and; (3) lack of compassion and understanding by service providers. The difficult healthcare environment had affected overall views and experiences of these women going on ART-for-life. All patient participants (n=55) responded they chose to take the FDC pill to protect the baby's health and believed lifelong ART could be stopped after delivery, unaware of the long-term benefits.

There are major challenges with the national guidelines. These changes affect the policies and procedures at RMMCH both at the clinical and management levels. Research studies tend to focus on the output of healthcare programming without including the user perspective before implementation. Therefore, by including pregnant HIV-positive women's experience of the Option B+ program is informative in understanding the patients' perception about going on ART for life to better understand strategies in implementing an adaptable approach for adherence.

## Strengths and Limitations

Policy often excludes the voices of those that are most impacted by its implementation. The strength of this study was its ability to provide a platform for pregnant HIV-positive women to share their lived experiences with lifelong ART. The study was able to explore the perceptions of HCWs from both clinical and management levels and how their work has been impacted by the national consolidated guidelines as a way to inform Option B+ PMTCT programming. Qualitative research develops concepts that can provide better understanding and speak to the concerns of those who provide healthcare and the interaction with their recipients (14, p. 42–45).

The use of body language of the PI may have affected the results through moderator bias. Therefore, acknowledgment and disclosure of the PI's perspective enhances the credibility of the study (15, p. 341–360; 17, p. 36–37). The use of triangulation strengthened the validation of the data in the study. In addition, because of the study's diverse and large sample, the study findings may be transferrable to other settings that are implementing this program.

This study focused on one hospital system in Johannesburg, South Africa, which limits the ability to make assumptions about the findings beyond the local context in which this study was conducted. Though the study was conducted six months after introduction to the Option B+ program, the study had delineated many issues earlier on that could be considered in shaping the program aspects, for example peer group support and knowledge transfer/education for HIV positive women.

Additionally, a convenience sample of HCWs was selected for qualitative interviews based on recommendations from the Head of the Pediatric and Child Health department and Director of the ESRU. In order to mitigate the limitation of biased selection of the HCWs, a purposeful random sampling method could be used to increase credibility of the study. To gain a maximum representation of views, ensuring that patients were not subjected to selection bias based on their ability to speak English, can increase the validity of the research.

## Recommendations and Conclusions

For lifelong treatment programming to function and be successful in other settings for HIV-positive pregnant women, certain components need to be addressed to ensure adaptability; by providing continuous counseling and support services for women with same day initiation.

RMMCH needs to improve their internal communication and collaboration amongst HCWs across all units to develop consistency for the use of indicators for the program and understanding of individual roles in delivering consistent messages and services to mothers. Communication is essential in helping patients build trust in service delivery, decreasing the LTFU and can alter the patient perception on long term adherence. Most importantly, an HIV-positive pregnant woman needs to understand the long-term benefits of FDC for both herself and her baby. This will help build trust in services utilized by these pregnant women and alter their perceptions on medication adherence holistically after delivery of the baby. A recommendation is for collaborative sessions that are feasible for HCWs with existent training sessions which can be managed with a rotation schedule for workers to come together and give their input.

A challenge for HCWs was the ability express sympathy for the patient. In highly constrained resource settings, a recommendation is to have support workshops for healthcare workers engaged in this work to manage and cope better. It is important to maintain the health of RMMCH?s staff. Support workshops can allow healthcare workers to come together and build closer relations to each other, understanding that they are all undergoing changes in their work.

Maintaining open communication and working on compassion and patience will make workers more aware that their attitude and behavior toward patients can impact the quality of care the patient receives. It is recommended that training workshops be held for frontline nurses and physicians.

It is crucial to understand the barriers to the short and long-term sustainability of the Option B+ program. Understanding the environment in which a PMTCT program operates can address policy implementation and program issues and hence inform how adaptable Option B+ programing is on a larger scale. The national guidelines have been developed but *adaptability* may remain problematic to successful roll out of the Option B+ program outside of RMMCH (11, p. 8–10).

Implications for this research include the need to address changes within the healthcare system at both clinical and management levels. It is crucial to incorporate the perspective of patients and frontline HCWs alike in policy implementation; uptake and adherence are key indicators in informing if the Option B+ PMTCT program is adopted into state hospitals effectively. This can help address issues before policy implementation is executed. Often, stakeholders and policymakers come together to create a policy to do what they see may be best from a top down approach. However, by using a community based approach, implementation plans can take place as a discussion by including the patient and healthcare workers at the table and spark open dialogue between the key actors and those impacted by the implementation. Community based approaches can empower individuals, such as these HIV-positive pregnant women to acknowledge expectations about themselves, their behavior and potential. The involvement of the patient living with the illness in the community can provide insight as to how Option B+ could be integrated and adapted to meet the unique needs and values of the surrounding community at healthcare facilities undergoing high resource constraints.

In addition, extensive research needs to be done on how to strengthen indicators for long term sustainability of the program in conducting a longitudinal study comparing system planning between healthcare facilities and state hospitals. Collaboration across the departments of RMMCH and can provide perspective and insight as to what indicators need to be strengthened for the Option B+ PMTCT program. This can be executed through interdisciplinary department meetings and training may encourage dialogue between staff internally and report any issues and concerns they may be experiencing with the program to upper management. RMMCH has existent clinical training sessions done specifically with frontline nurses. By including the physicians and counselors that work within the departments, this can overall strengthen the communication between HCWs. Remember all HCWs can provide feedback as to how their work environment is managing these indicators, which may be entirely different depending on which department they work in. Future evaluations need to address if interdisciplinary collaboration within hospitals can improve the management and understanding of Option B+.

Furthermore, many pregnant women were interested in learning more about the ARVs they were on, especially FDC and why they had to take it. Limited information is provided from the education that nurses give in the antenatal clinic. It is recommended that instead of delivering education and information in a lecture style format, it should be conducted as interactive sessions to stimulate the patients and keep them engaged. Interactive sessions allow pregnant HIV-positive women to build mentorship and a network where they can form healthy relationships which can improve their adherence to ART (16).

Womens' perceptions of their partners' approval of their HIV status have been identified as a strong predictor of willingness for women to return to the antenatal clinic (18). RMMCH provides women with counseling but often males are not allowed into the facility which acts as a barrier to uptake. A recommendation is to provide additional support groups where the male partners can attend to increase open communication by including the male in child spacing and family planning methods.

This research will contribute to the HIV/AIDS prevention program literature in the health policy field. In our increasingly globalized world, it is important to determine how processes and policies in one country can be successfully adapted to another country (11, p. 8–10).

## Data Availability Statement

The datasets for this manuscript are not publicly available to protect confidentiality and HIV status for participants. Requests to access the datasets should be directed to Melanie A. Bisnauth, melaniebisnauth@gmail.com.

## Ethics Statement

The studies involving human participants were reviewed and approved by The Hamilton Integrated Research Ethics Board (HiREB) at McMaster University located in Hamilton, Canada and the Human Research Ethics Committee (HREC) at the University of Witwatersrand. Written informed consent to participate in this study was provided by the participants/participants' legal guardian/next of kin.

## Author Contributions

The authors retained full control of the primary data. The content of the research in this document has been completed by MB and recognizes the contributions of the authors. This study benefited from the participation of a wide range of partners, medical professionals, HIV specialists and researchers. The author is grateful for the guidance provided by the committee members for manuscript management. All members read, commented on, and approved the final manuscript.

## Conflict of Interest

The authors declare that the research was conducted in the absence of any commercial or financial relationships that could be construed as a potential conflict of interest.
